# The impact of higher education on high quality economic development in China: A digital perspective

**DOI:** 10.1371/journal.pone.0289817

**Published:** 2023-08-09

**Authors:** Bao Bing

**Affiliations:** School of International Business and Economics, Changchun University of Finance and Economics, Changchun, China; Zhejiang Gongshang University, CHINA

## Abstract

In the context of China’s new stage of economic development, this study examines the role of higher education in China’s high quality economic development (HQED) strategy from a digital perspective. Using panel data of 30 Chinese provinces (municipalities and autonomous regions) collected from 2012–2020, comprehensive evaluations of the level of higher education and HQED are conducted through the entropy method, and a regression analysis is carried out with the fixed effect model. The results show that the level of higher education is positively associated with HQED and is able to achieve this effect through mechanisms that actively promote digital innovation and development. Further, the structure and quality of higher education plays a greater part in facilitating digital development than the scale and quantity. The heterogeneity analysis demonstrates that the impact of higher education on HQED is more significant in the eastern region of China than in the western region. An increase in the proportion of fiscal expenditure to GDP diminishes the impact of higher education on HQED, while an improvement in digital governance level enhances its influence.

## 1. Introduction

The impact of higher education on economic development has become a topic of increasing interest in both academic and policy circles [1]. The variation in educational participation contributes to differences in economic performance among countries [2]. According to *China Education Daily*, China’s gross enrolment rate in higher education increased from 30% in 2012 to 57.8% in 2021—an increase of 27.8 percentage points—achieving a historic leap forward in building the world’s largest higher education system. Meanwhile, China’s economy has achieved rapid, but consistent, growth since it commenced its policy of economic reform and opening-up in the late 1970s. The popularization of higher education enhances its crucial role in facilitating economic growth [3].

In recent years, China is focused on shifting from prioritizing high-speed growth to a stage of high-quality development. This approach, put forward for the first time in 2017, is designed to better stimulate the endogenous dynamics of China’s economic development. Consequently, it becomes crucial to examine the impact of China’s higher education system on high-quality economic development (HQED) in light of the new environment and dynamics.

Some researchers have conducted investigations into higher education in the context of high-quality personnel training as crucial support for socioeconomic development. Schultz’s [4] theory of human capital documented, for instance, is generally accepted as the seminal foundation upon which this subject has been explored. Human capital is an important factor in economic growth, and education plays a much greater role in human capital than social training, health care [5], or other prevalent social systems. The effect of human capital promotion, which arises from educational expansion [6], graded education [7], and supply-side structural education reform [8], on economic development is significantly positive [9, 10]. More universities in a region lead to stronger economic growth, as research quality and academic specialization become the main drivers of universities’ positive impact on regional economic development [11].

Numerous models and methods have been applied to study the relationship between higher education and economic development. Granger methods, panel data models, and two-stage regression on a modified Cobb–Douglas production function are among the techniques used to test the causal relationship between higher education and economic growth [12–14]. Macroeconomic regression studies also support the positive impact of higher education on regional economic development and social cohesion [15]. However, the existing quantitative research on the relationship between higher education and economic development is relatively insufficient, necessitating a more comprehensive and multidimensional assessment of them.

The literature does present divergent views, suggesting that, while education is important for economic development, it is not a sufficient condition. Limited higher education supply can promote economic growth by increasing overall savings, but when this growth-promoting effect becomes strong enough, it leads to a shortage of investable capital, thereby reducing economic growth [16]. There are significant variations in the interplay between the characteristics of higher education and economic performance across different levels of economic development [17]. To formulate effective policies, there is a need for a comprehensive understanding of the relationship between higher education and the economy [18], particularly during the new phase of HQED.

A number of studies have explored the mechanisms by which higher education impacts economic development. Education cannot only generate the accumulation of human capital, but can also promote technological progress, which is the key to stimulating economic growth [19]. Similarly, higher education can also facilitate economic development by enhancing innovation capacity, optimizing industrial structure, and promoting new urbanization [20–23]. With the rapid advancement of information technology, digitalization has become crucial for modern economic development. Integrated digital infrastructure and technologies drive industrial transformation [24, 25], digital finance enhances ecological governance, and the digital economy fosters increased tax revenue [26] and promotes shared prosperity [27]. These factors play a vital role in ensuring HQED in the digital era. Nonetheless, there is limited research that addresses the mechanism through which higher education can influence HQED by digital advancements.

This study seeks to examine the impact of higher education on HQED in China from the digital perspective, using the entropy evaluation method and fixed effects model with panel data collected from 2012–2020. As further robustness checks, two alternative methods, principal component analysis and standardization, are utilized to evaluate the level of higher education and HQED. Heterogeneity analysis was conducted based on the regional perspective, temporal dimension, fiscal expenditure level, and digital governance level. The impact of higher education on HQED was assessed from the relatively new perspective of digital development. This provides new insights into the mechanism of higher education’s influence on economic development, and will provide an important reference for the formulation and implementation of education policies to advance economic development.

## 2. Background and theoretical framework

### 2.1 Background: China’s new development philosophy

Based on previous experience gained and lessons learned both at home and abroad, alongside analysis of the current trend of global development, China has forged a new development philosophy of pursuing innovative, coordinated, green and open development, and having its fruits shared by everyone. This is intended to target problems that have been present in China’s development model since 2012. It is focused on shifting from a period of high-speed growth to a high quality development. Since this concept with Chinese characteristics was put forward for the first time in 2017, researchers in China have begun to turn to the study of high quality development, mainly discussing its cultural connotations [28, 29] and contributing factors [30]. Some have also focused on the relationship between environment [31], technological innovation [32], and HQED. This study seeks to discover what impact higher education will have on HQED in this new phase.

### 2.2 Theoretical framework

In the increasingly complex global economic landscape, human capital is one of the main pillars driving high-quality and sustainable economic growth [33]. As the core of human capital, higher education bears primary responsibility for advanced talent training and development of scientific research capabilities and is a cardinal tool for sustainable economic growth and development [34]. Some studies have shown that education affects economic growth both directly and indirectly by driving industrial upgrading, regulating income distribution, promoting consumption [35] and technological innovation [20, 21]. Hence, the first hypothesis is proposed as follows:

**Hypothesis 1**. Higher education has a positive impact on HQED.

**Fig 1:**
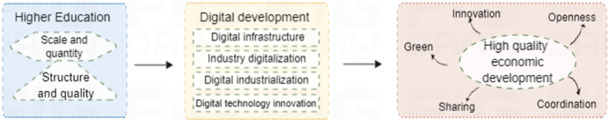
Theoretical analysis framework.

Higher education provides specialized training through the construction of digital-related disciplines, which, in turn, provide enterprises with digitally competent employees and contractors, who become a key support for the development of digital industries. In the course of governing and serving society, the dissemination of digitized knowledge and information through higher education becomes the basis for a vibrant digital economy. The transformative digital technological achievements of higher education enable new companies to develop and digital production to function soundly, implying that it is undoubtedly one of the key drivers of digital development. The digitization of education, exemplified by the use of shared digital learning space [36] and virtual classrooms [37], is also an important part of digital development. Based on these vital contributions, it is logical that higher education should have a positive impact on digital development. In this process, we must not disregard the "quantity" and "quality" of education. From a quantitative perspective, the scale of higher education institutions and the number of students will affect the stock of human capital, which, in turn, shapes the capacity for knowledge innovation and increases the potential for digital technological progress. As digital technology continues to develop, how does it affect the relationship between higher education and economic development? Fig 1 illustrates the theoretical framework employed in this paper.

Meanwhile, the differences in the structure and quality of higher education, such as the composition of postgraduate and undergraduate levels, the proportion of teachers with senior titles, and the capacity for R&D, are key factors that influence the level of human capital available. These resources form the basis for technological transformation and upgrading, which can contribute to the rapid development of digital industries.

Some studies have focused on the contribution of information and communications technologies (ICT) and digital development to economic growth and productivity [38, 39]. From a dynamic long-term perspective, digital-related industries will have a positive impact on the economy. Artificial intelligence (AI), for example, will lead to an increase in the economic productivity of the e-commerce industry [40] and new digital infrastructure can drive high quality regional economic development [41]. The digital economy has indeed positively promoted China’s green total factor productivity [42] through industrial restructuring and green technology innovation. It also drives high quality urban development by improving the efficiency of physical capital allocation [36]. This suggests that we can expect a mechanism whereby higher education contributes to the quality of the overall economy by promoting digital development. Thus, the second and third hypotheses are as follows:

**Hypothesis 2**. Higher education stimulates the level of high-quality economic activity through digital development.
**Hypothesis 3**. Both the “scale and quantity” and the “structure and quality” of higher education contribute to digital development.

## 3. Evaluation index system and data

### 3.1 Construction of evaluation index system

In this paper, educational scale and quantity along with structure and quality are considered to represent the level of higher education development with nine indexes selected (Table 1). Based on the available literature, with full consideration of China’s actual development, a comprehensive index system for assessing the level of HQED from the perspective of China’s new development philosophy is established [43]. HQED is measured by five criteria from the new development philosophy, which are innovation, coordination, green, openness, and sharing, including 20 indexes. The criteria for assessing the digital development level were digital infrastructure, industry digitalization, digital industrialization, and digital technology innovation. The index system is shown in Table 1.

**Table 1:** Indicator of higher education, HQED and digital development.

System layer	Criterion layer	Index layer	Index attribute
Higher education	Scale and quantity	Number of regular higher educational institutions (unit)	+
		Average number of students enrollment in higher education per 100,000 population (person)	+
		Number of students in general universities (10,000 persons)	+
		Number of educational personnel in regular higher educational institutions (10,000 persons)	+
	Structure and quality	Student–teacher ratio (number of teachers = 1)	-
		Number of full-time teachers with senior titles (10,000 persons)	+
		Number of graduate students (person)	+
		Intramural expenditure on R&D in higher education (10,000 yuan)	+
		Full-time equivalent of R&D personnel (man-year)	+
HQED	Innovation	The R&D expenditure input intensity (%)	+
		Full-time equivalent of R&D personnel (man-year)	+
		Number of three kinks of domestic patents granted (inventions, utility models and designs) (piece)	+
	Coordination	Industrial rationalization: Theil index	-
		Industrial advancement: value-added of the tertiary industry/ value-added of the secondary industry	+
		Urban–rural per capita disposable income ratio	-
		Urban–rural per capita consumption ratio	-
		Registered unemployment rate in urban areas (%)	-
	Green	Energy consumption per unit of output (10, 000 tce/100 million yuan)	-
		Volume of COD discharged (10,000 tons)	-
		Volume of sulfur dioxide emission (10,000 tons)	-
		Green covered area as percentage of built-up area (%)	+
		Energy conservation and environmental protection expenditure/fiscal expenditure	+
	Openness	Total value of trade in goods/ GDP	+
		Total investment of foreign invested enterprises (million US dollars)	+
		Foreign exchange earnings from international tourism (million US dollars)	+
	Sharing	Government expenditure on social security and employment (100 million yuan)	+
		Length of highways (10,000 km)	+
		Expenditure for education (100 million yuan/10,000 persons)	+
		Expenditure for health (100 million yuan/10,000 persons)	+
Digital development	Digital infrastructure	Popularization rate of mobile telephone (sets/100 persons)	+
		Length of optical cable lines (km)	+
		Broad band subscribers port of Internet (10,000 ports)	+
	Industry digitalization	Degree of digitalization of digital financial inclusion	+
		E-commerce sales (100 million yuan)	+
	Digital industrialization	Total telecom business (100 million yuan)	+
		Software income (100 million yuan)	+
	Digital technology innovation	Internal expenditure on R&D expenses in the electronics and communications equipment manufacturing industry (10,000 yuan)	+

### 3.2 Data source

Statistical data from 2012 to 2020 required for this study were primarily obtained from the China Statistical Yearbook, the China Statistical Yearbook on Science and Technology, the Educational Statistics Yearbook of China, the China Energy Statistical Yearbook, China Statistical Yearbook on High Technology Industry, the statistical yearbooks of the provinces, the website of the National Bureau of Statistics of China (http://www.stats.gov.cn/), and the Ministry of Education of the People’s Republic of China (http://www.moe.gov.cn/). The digital financial inclusion index of China is derived from the Institute of Digital Finance Peking University (https://idf.pku.edu.cn/). Some missing data, which are less than 1%, are supplemented by linear interpolation. Data entry and some calculations, such as the energy consumption per unit of output, the intensity of energy conservation and environmental protection (energy conservation and environmental protection expenditure/fiscal expenditure), and the level of financial development (financial industry value added/GDP), were executed in Excel 2010. Descriptive statistical analysis was conducted using SPSSAU. The Tibet, Hong Kong, Macao, and Taiwan regions were excluded from this analysis for statistical reasons.

### 3.3 Variable evaluation and description statistics

To evaluate the level of HQED, the original data were first standardized to eliminate the dimensional differences according to the positive and negative directions. Based on the above construction of the evaluation index system, a composite index was calculated using the entropy evaluation method, in which the evaluation result is free from the influence of human subjectivity. This approach is also applied in this study when measuring the level of higher education and digital development.

**Fig 2:**
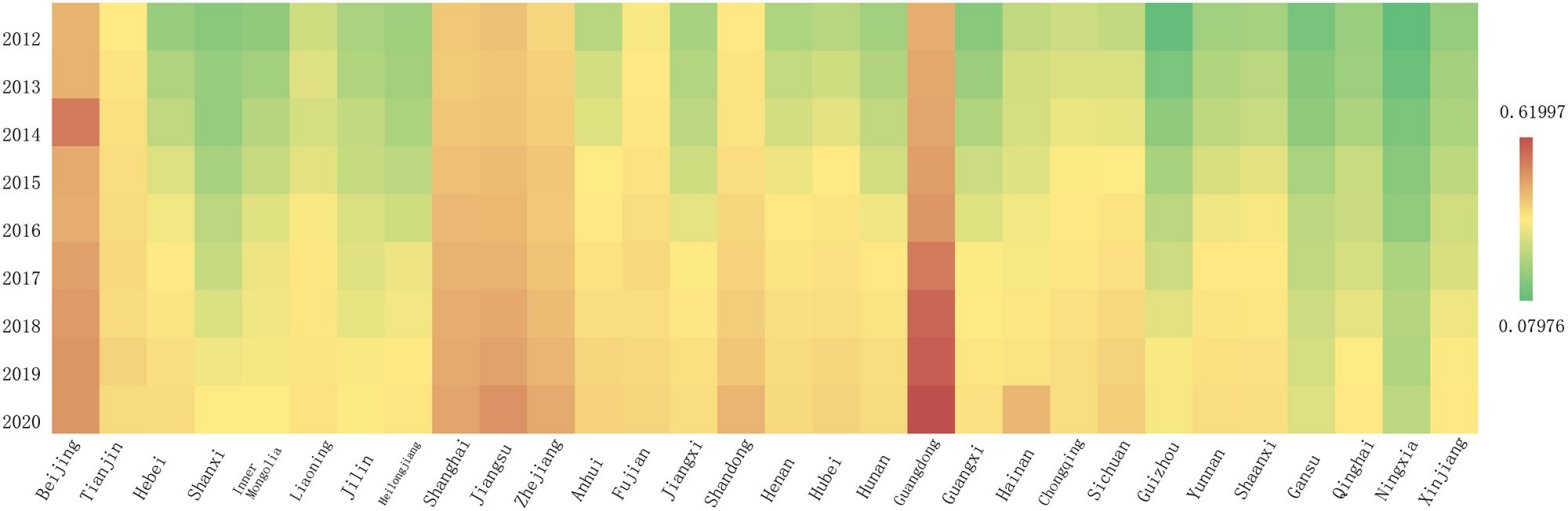
Level of HQED.

**Fig 3:**
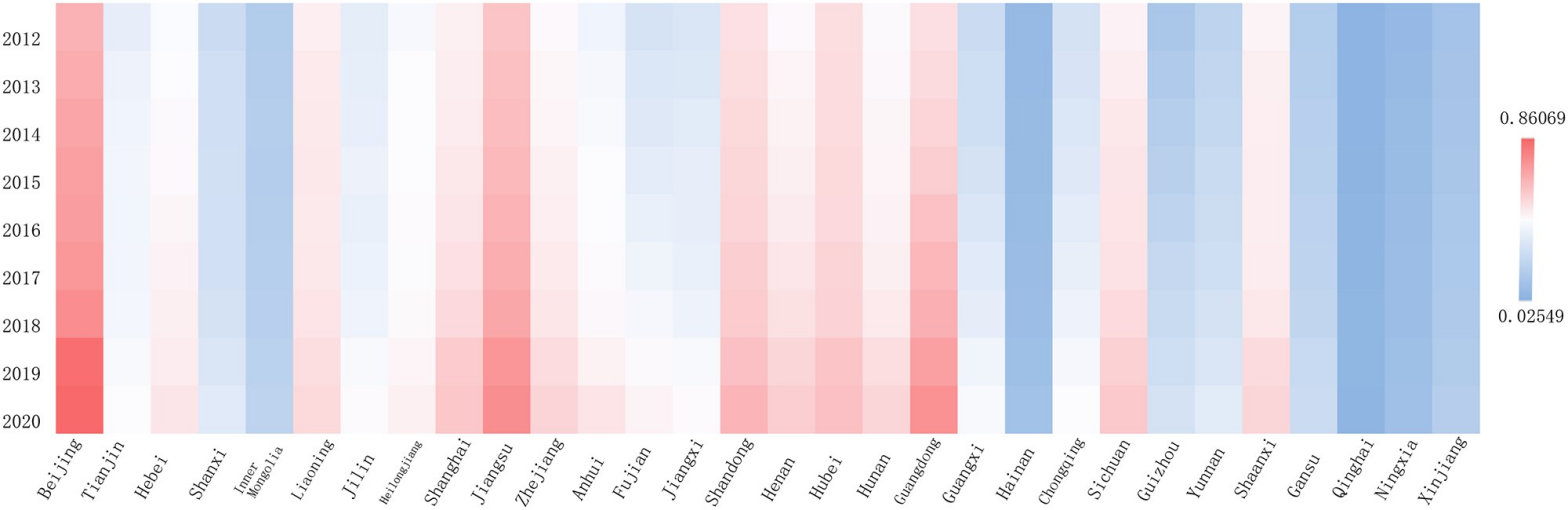
Level of higher education development.

The economic development level calculated accordingly is shown in Fig 2. In 2012, only two provinces (Beijing and Guangdong) had a HQED composite score of 0.3 or above. However, in 2020, the number had increased to seven provinces. Additionally, in 2012, three provinces’ composite scores were lower than 0.1, while in 2020, the lowest level had been raised to 0.13 (Ningxia). Fig 3 indicates the higher education level in China in 2012 and 2020. Compared with the level of HQED, the level of higher education development is relatively high, with the composite score of 0.5 and above expanding from Beijing to Jiangsu, Hubei, Shandong, and Guangdong. The statistical description is shown in Table 2.

**Table 2:** Statistical description of major variables.

Variables	Mean	Standard deviation	Min	Max	Observations
HQED	0.190	0.089	0.080	0.620	270
Higher education	0.276	0.160	0.025	0.861	270
Scale and quantity	0.414	0.201	0.011	0.878	270
Structure and quality	0.208	0.157	0.023	0.983	270
Digital development	0.150	0.134	0.021	0.943	270
Marketization degree	0.273	0.121	0.123	0.999	270
Industrialization degree	8.850	1.188	5.814	10.976	270
Level of financial development	0.071	0.032	0.026	0.196	270
Level of fiscal expenditure	0.253	0.104	0.118	0.643	270

## 4. Empirical results

### 4.1 Econometric model

The following econometric model was used to estimate the impact of higher education on HQED:

$$HQED_{it}=\beta_{0}+\beta_{1}EDU_{it}+\beta_{i}X_{it}+\lambda_{i}+\eta_{t}+\mu_{it}$$ (1)

The dependent variable, *HQED*_*it*_, is an indicator of the economic development quality level of region *i* in year *t*. The key independent variable, *EDU*_*it*_, is an indicator of the degree of higher education in region *i* in year *t*. *λ*_*i*_ denotes the region fixed effect, while *η*_*t*_ represents the time fixed effect, and *μ*_*it*_ is a disturbance term. The degrees of marketization and industrialization and the level of financial development, which are characterized as the proportion of state-owned and collective components in the total social fixed asset investment portfolio, the natural logarithm of the number of industrial enterprises above designated size, and financial industry value added to GDP ratio respectively, serve as control variable, *X*_*it*_.

In addition, to test the role of digital development, the model was constructed as follows:

$$DIG_{it}=\gamma_{0}+\gamma_{1}EDU_{it}+\gamma_{i}X_{it}+\lambda_{i}+\eta_{t}+\mu_{it}$$ (2)

The level of digital development is described by DIG_it_. The other variables included are identical to those in Eq (1).

### 4.2 Results

Multicollinearity analyses revealed that variance inflation factors were smaller than 4 for all predictor variables (1.358–3.410), confirming that regression models were not affected by the presence of multicollinearity. The choice of a fixed effects specification was supported by the Hausman test.

Table 3 illustrates the impact of higher education on HQED. The effects presented in Column 1 suggest that a 1-unit increase in the level of higher education corresponds to an increase in HQED of 0.418 points. After including control variables, the estimate result drops to 0.415 in Column 2 and remains statistically significant.

**Table 3:** The impact of higher education on HQED.

	Dependent variable: Level of HQED					
	Calculated by entropy method	Calculated by entropy method	Calculated by PCA	Calculated by PCA	Standardized treatment	Standardized treatment
	1	2	3	4	5	6
Level of higher education	0.418**	0.415**	0.203**	0.190**	0.755**	0.748**
	(9.513)	(9.554)	(5.420)	(5.229)	(9.513)	(9.554)
Control variables	No	Yes	No	Yes	No	Yes
Provincial fixed effect	Yes	Yes	Yes	Yes	Yes	Yes
Year fixed effects	Yes	Yes	Yes	Yes	Yes	Yes
*R*^2^(within)	0.577	0.590	0.199	0.090	0.577	0.590
Observations	270	270	270	270	270	270

Note: The original data are from the China Statistical Yearbook, the China Statistical Yearbook on Science and Technology, the Educational Statistics Yearbook of China, the China Energy Statistical Yearbook, China Statistical Yearbook on High Technology Industry, the Statistical Yearbooks of provinces, the website of the National Bureau of Statistics of China, and the Ministry of Education of the People’s Republic of China. Control variables include the marketization degree, the industrialization degree and the level of financial development. The t-values are in parentheses.

* p<0.05,

** p<0.01.

On this basis, an alternative method was used to measure the independent and dependent variables; the results remained robust. Columns 3 and 4 in Table 3 report the estimates using principal component analysis (PCA) to calculate the corresponding levels of higher education and HQED. The estimates show that a 1-unit increase in the level of higher education corresponds to an increase of 0.203 and 0.190 points without and with control variables, respectively. Although the estimated regression coefficients in Columns 3 and 4 are much lower compared to Columns 1 and 2, they are still statistically significant. These findings suggest that higher education is significantly associated with HQED.

Another robustness test was also performed using the standardized method. After calculating the level of higher education and HQED using the entropy evaluation method, the consequences were standardized using Eq (3).


$$z_{i}=\frac{x_{i}-\bar{x}}{s},\;s=\sqrt{\frac{1}{N-1}\sum_{i=1}^{N}{\left(x_{i}-\bar{x}\right)}^{2}}$$ (3)

The regression results are reported in Table 3, Columns 5 and 6. The estimates show that a one standard deviation increase in the level of higher education is associated with a 0.755 standard deviation increase in HQED in Column 5. With control variables, the same change leads to an increase of 0.748 standard deviations in HQED in Column 6. This reveals that the regression results are still significant after the values have been standardized, further confirming the previous finding that higher education has a significant impact on the quality of economic development.

### 4.3 Mechanisms: Digital development

The above analysis has proved robustly that China’s higher education level significantly promotes HQED. I will now continue to discuss the mechanism.

Higher education is enabling digital development. This channel is verified through Eq (2). The regression results reported in Columns 1 and 2 of Table 4 are 1.491 and 1.492, showing that the estimation coefficients are significantly positive with and without control variables, which demonstrates that improving the level of higher education can facilitate advancement of digital development. Columns 3–4 and 5–6 in Table 4 report the results of regressions using PCA to calculate the level of higher education and HQED, and regressions using standardized consequences through Eq (3), respectively. These results further validate the significant impact of higher education on digital development.

**Table 4:** The impact of higher education on digital development.

	Dependent variable: Level of digital development (DIG)					
	Calculated by entropy method	Calculated by entropy method	Calculated by PCA	Calculated by PCA	Standardized treatment	Standardized treatment
	1	2	3	4	5	6
Level of higher education	1.491**	1.492**	1.373**	1.372**	1.782**	1.783**
	(28.318)	(28.389)	(19.088)	(19.556)	(28.318)	(28.389)
Control variables	No	Yes	No	Yes	No	Yes
Provincial fixed effect	Yes	Yes	Yes	Yes	Yes	Yes
Year fixed effects	Yes	Yes	Yes	Yes	Yes	Yes
*R*^2^(within)	0.894	0.875	0.726	0.603	0.894	0.875
Observations	270	270	270	270	270	270

Note: Control variables include the marketization degree, the industrialization degree and the level of financial development. The t-values are in parentheses.

* p<0.05,

** p<0.01.

Digitalization also drives HQED. As outlined in the theoretical framework section of this paper, previous studies have confirmed that digital-related industries such as new digital infrastructure, ICT, and AI can drive HQED [38–42, 44]. The above mechanisms demonstrate that the level of higher education significantly contributes to digital progression, and thus to HQED.

What impact will different aspects of higher education have on digital development? According to the evaluation index system presented in this study, the contribution of higher education to digital development will be measured in terms of scale and quantity, as well as structure and quality. Column 1 in Table 5 reports that a 1 standard deviation increase in higher education correspondents to a 1.149 standard deviation increase in the level of digital development. This compares with an increase of 1.157 when control variables are included, as shown in Column 2, Table 5. The results in Columns 3–4 suggest that structure and quality in higher education also have a significantly positive impact on digital development (1.206, 1.228); obviously, the impact of scale and quantity of higher education is slightly smaller by comparison.

**Table 5:** The influence of different factors in higher education on digital development.

	Dependent variable: Level of digital development (DIG)			
	Scale and quantity		Structure and quality	
	1	2	3	4
Sub-criteria of higher education	1.149**	1.157**	1.206**	1.228**
	(6.870)	(6.420)	(22.389)	(23.736)
Control variables	No	Yes	No	Yes
Provincial fixed effect	Yes	Yes	Yes	Yes
Year fixed effects	Yes	Yes	Yes	Yes
*R*^2^(within)	0.459	0.485	0.793	0.719
Observations	270	270	270	270

Note: The calculation method of independent and dependent variables: after calculating the complex scores of higher education and digital development using the entropy evaluation method, consequences thereof are standardized through Eq (3). Control variables include the marketization degree, the industrialization degree, and the level of financial development. The t-values are in parentheses.

* p<0.05,

** p<0.01.

### 4.4 Heterogeneity analysis

#### 4.4.1 Regional heterogeneity analysis

The economy is evolving, but not equally across different regions. According to information obtained from the National Bureau of Statistics, China’s economic zones are divided into the Eastern, Central, Western, and Northeastern regions to reflect different regional economic development dynamics. Therefore, regional heterogeneity can be better reflected by defining variables at the Four Regional levels. Higher education development and HQED were measured at the four regional levels to reexamine the relationship between the two. Using the western region as the reference group, the results, reported in Table 6, indicate that, although the degree of influence decreased, the Eastern Region’s higher education level had a greater impact on HQED than that of the western region. This may be because the eastern region is rich in education resources and can thus provide higher-end talent support.

**Table 6:** Results of regional heterogeneity.

	Dependent variable: Level of HQED			
	1	2	3	4
Higher education	0.068**	0.033**	0.040**	0.024**
	(19.956)	(5.091)	(7.393)	(3.188)
Higher education × eastern region		0.038**		0.030**
		(4.923)		(4.009)
Higher education × central region		0.011		0.012
		(0.868)		(1.043)
Higher education × northeastern region		0.012		-0.003
		(0.461)		(-0.120)
Control variables	No	No	Yes	Yes
*R* ^2^	0.598	0.759	0.711	0.786
Observations	270	270	270	270

Note: China’s economic regions are divided into the Eastern, Central, Western and Northeastern Regions according to information obtained from the National Bureau of Statistics. The western region was used as the reference group. The Tibet, Hong Kong, Macao, and Taiwan regions were excluded from this analysis for statistical reasons. Control variables include the marketization degree, the industrialization degree and the level of financial development. The t-values are in parentheses.

* p<0.05,

** p<0.01.

#### 4.4.2 Temporal heterogeneity analysis

Digital development as a means of driving changes in production methods has become an important theme of economic advancement in the new era. Policy documents related to digital development have served as the main source of guidance. The Digital China initiative, launched in December 2015, marked the transition of digital development from the initial stage of exploration to comprehensive construction. After 2016, policies such as the 13th Five-Year Plan for National Informatization and the 14th Five-Year Plan for the Development of Deep Integration of Informatization and Industrialization were proposed. I therefore use 2015 as the cut-off point for indirect analysis of the data for the two intervals 2012–2015 and 2016–2020, as shown in Table 7. The regression results demonstrate significant results between 2012 and 2015 with or without the inclusion of control variables. The development of higher education from 2016 and 2020 is shown to have had significant positive impacts on HQED. It is not apparent from the results whether the promotion of higher education and the support of digital-related policies exerted a superimposed and multiplier effect on the digital economy, thus contributing to the high-quality development of the economy. It needs to be examined over a longer period of time.

**Table 7:** Results of temporal heterogeneity.

	Dependent variable: Level of HQED			
	1: 2012–2015	2: 2012–2015	3: 2016–2020	4: 2016–2020
Higher education	0.984**	1.070**	0.670**	0.664**
	(2.707)	(2.844)	(5.338)	(5.439)
Control variables	No	Yes	No	Yes
Provincial fixed effect	Yes	Yes	Yes	Yes
Year fixed effects	Yes	Yes	Yes	Yes
*R* ^2^	0.352	0.417	0.525	0.557
Observations	120	120	150	150

Note: Using 2015 as the cut-off point for indirect analysis through policies of digital development. Control variables include the marketization degree, the industrialization degree, and the level of financial development. The t-values are in parentheses.

* p<0.05,

** p<0.01.

#### 4.4.3 Heterogeneity analysis based on fiscal expenditure level

Restructuring fiscal spending is an important factor in economic growth [45]. Therefore, I also examine the differences in varying scenarios of fiscal expenditure levels. The level of fiscal expenditure is measured by the ratio of fiscal expenditure to GDP. Regions were classified into high fiscal expenditure and low fiscal expenditure based on a threshold of 0.253 (Table 2) for the moderation tests. Compared with regions with low-level fiscal expenditure, the results reported in Table 8 indicate that an increase in the proportion of fiscal expenditure to GDP weakens the impact of higher education on the high-quality development of the economy. This could be due to the excessive reliance on fiscal subsidies, which may diminish the autonomy and independence of higher education, undermine its ability to adapt to market demand, and affect its capacity to serve and innovate for the economy. This could result in a reduction of market orientation and competitive mechanisms, as well as give rise to issues of resource allocation efficiency.

**Table 8:** Results of heterogeneity analysis based on fiscal expenditure level.

	Dependent variable: Level of HQED			
	1	2	3	4
Higher education	0.068**	0.088**	0.040**	0.050**
	(19.956)	(18.980)	(7.393)	(10.250)
Higher education × fiscal expenditure level		-0.076**		-0.080**
		(-5.694)		(-6.924)
Control variables	No	No	Yes	Yes
*R* ^2^	0.598	0.655	0.711	0.777
Observations	270	270	270	270

Note: Regions were classified into high and low fiscal expenditure based on a threshold of 0.253. The group of low fiscal expenditure regions was used as the reference group. The Tibet, Hong Kong, Macao, and Taiwan regions were excluded from this analysis for statistical reasons. The t-values are in parentheses.

* p<0.05,

** p<0.01.

#### 4.4.4 Heterogeneity analysis based on digital governance

Digital governance enables the integration of policies and public services through e-government, fostering sustainable and inclusive economic growth and social development [46]. The Digital Government Development Index, as defined in the “2020 Digital Government Development Index Report” by the Center on Data Governance, Tsinghua University, serves as a measure of the level of digital governance. I categorize the eight provinces highlighted as “leading” and “high quality” in the report as regions with a high level of digital governance, while the remaining provinces are classified as low digital governance regions. The results of moderation tests in Table 9 show that improved levels of digital governance enhance the impact of higher education on high-quality economic development. Digital transformation enables higher education to utilize information technology more effectively, providing high-quality teaching resources and innovative platforms, and offering professional and intellectual support for digital government development. Simultaneously, digital administrative operations and data sharing enable governments to better meet market demands as well as promote effectiveness and fairness in resource allocation. This mutually reinforcing relationship contributes to driving high-quality economic development.

**Table 9:** Results of heterogeneity analysis based on digital governance.

	Dependent variable: Level of HQED			
	1	2	3	4
Higher education	0.068**	0.042**	0.040**	0.019**
	(19.956)	(10.268)	(7.393)	(2.888)
Higher education × digital governance		0.044**		0.042**
		(6.572)		(5.485)
Control variables	No	No	Yes	Yes
*R* ^2^	0.598	0.705	0.711	0.753
Observations	270	270	270	270

Note: According to the “2020 Digital Government Development Index Report,” eight provinces, labeled as “leading” and “high quality,” are designated as high-level regions of digital governance. The remaining provinces are considered low-level regions, and comprise the reference group. The Tibet, Hong Kong, Macao, and Taiwan regions were excluded from this analysis for statistical reasons. The t-values are in parentheses.

* p<0.05,

** p<0.01.

## 5. Conclusion and discussion

This article examines the impact of Chinese higher education on HQED and its mechanisms from a digital perspective, using panel data from 30 Chinese provinces from 2012 to 2020. The main conclusions are as follows.

First, the results of the benchmark regression show that the advancement of China’s higher education can significantly contribute to HQED, which is confirmed in the robustness tests. The regression results verify theoretical hypothesis 1, and is consistent with previous studies [9, 34, 47]. New economic theory identifies investment in education and human capital as determinants of economic growth. Studies based on Keynesian economic principles and human capital theory have found that the expansion of higher education in China has brought economic growth effects and human capital development benefits [48]. Further, the optimization and improvement of the higher education personnel training structure has enhanced the workforce’s skills and become an important driver for the development of China’s emerging industries, contributing to the transformation and upgrading of the economy.

Second, the mechanism test proves that higher education contributes to a high-quality economy at a national level through the promotion of digital development, and that the role of higher education is not only reflected in the expansion of scale and quantity, but also because of structural and quality improvements. This also validates theoretical hypotheses 2 and 3 of this study. I speculated that more higher education institutions, more students in general universities, and more educators might all contribute to a more advanced digital economy. China officially entered the stage of massification and generalization of higher education with a gross enrolment rate of over 15% in 2012 and 50% in 2019, respectively [49]. The expansion of student enrolment, the increase in the number of schools, and the increased variety of educational disciplines over time have laid the foundation for the long-term sustainable development of higher education [50] and have, in turn, provided the necessary foundations for digital development. These regions give full play to the advantages of universities in the field of digital transformation, science and technology, and have opened digital economy-related majors, such as Internet of Things, virtual reality, and other emerging technologies, forming a disciplinary layout that strongly adapts to the digital development [51]. Universities provide strong intellectual support for the rapid and sustainable development of regional digitalization, and can better meet the needs of the digital industry development. They also serve as important bases for academic research and are able to achieve digital technology innovation directly through key technological research [52, 53]. In addition, the “structure and quality” of higher education plays a greater role with regard to digital development than the “scale and quantity.” Therefore, measures such as the training of high-level talents and the increase of R&D related investments are necessary.

Third, the contribution of higher education to quality economic development varies in different regions of China. The regional heterogeneity exhibited that higher education levels have a more positive influence on HQED in the east of China than in the west, which is consistent with existing research findings [54, 55]. Since education resources are more abundant in the east [56], and technological achievements are also generated more quickly because this region is at the forefront technological development—all of which makes it easier to upgrade the level of higher education in this region; especially in the eastern region, as exemplified by cities such as Beijing, Shanghai and Guangdong, where the ease of promoting the development of digital industries has directly contributed to the high quality of economic development present in these areas. This phenomenon may also be related to the fact that higher education talents from the western region are seen as preferable to the economically developed coastal regions, or to the civil service and institutions, which is primarily because people in less economically developed areas are culturally more inclined to pursue more stable jobs. Therefore, policies should be tailored according to the individual regions that they are intended to support. For example, to achieve the same effect in the Western and Eastern Regions would require a greater level of investment in higher education in the West. In addition, the test for temporal heterogeneity did not yield significant results. Perhaps because digital-related policies require a longer time to manifest the effects, or because of the dual negative impact of the COVID-19 pandemic on education [57] and the economy.

Taking it a step further, based on an analysis of fiscal expenditure level and digital governance, I found that an increase in the proportion of fiscal expenditure to GDP weakens the impact of higher education on HQED. This may reduce market orientation and competition mechanisms, ultimately affecting resource allocation efficiency. Therefore, policymakers must carefully consider structural adjustments to fiscal expenditure, which will help promote greater autonomy and innovation within higher education institutions, further supporting HQED. In contrast, an improvement in digital governance enhances the impact of higher education on HQED. Digital governance enables better digital administrative operations and data sharing, allowing governments to better meet market demands and promote effectiveness and fairness in resource allocation. Policymakers and stakeholders should prioritize investments in digital infrastructure and administrative capabilities to support the integration of higher education with digital government initiatives.

These findings verify the positive impact of higher education on HQED, which enriches the existing research on the relationship between higher education and economic development. Moreover, the introduction of digital development factor lends a new perspective to the study. Using China’s new development concept to construct an evaluation index system is suitable for the characteristics of the economic development stage and expands the ideas for regional economic assessment.

These conclusions have important practical implication for Chinese provinces to implement the coordinated development of higher education and the economy as a whole with the further advancement of digital development. It is conducive for local governments to understand the status of the relationship between higher education and economic development at the current stage, providing planning guidance and differentiated policy formulation according to the reality of the two systems. It is beneficial to leverage digital development to transform the advantages of higher education into a support guarantee for HQED through the cultivation of digital talents, increasing investment in educational innovation and effective expansion of the scale of education, and promoting a positive interaction pattern between higher education and HQED.

## 6. Limitations and future work

This study demonstrates a strong relationship between higher education and HQED, which has been verified through a number of robustness checks. From the perspective of digital development, the findings provide useful insights and can be considered enlightening. This study provides some foundation evidence for future studies. However, limitations remain. First, this study did not cover a longer period due to limitations in the data available on digital evaluation. In the future, it is possible to examine over a longer period of time whether higher education and digital related policies have a superimposed and multiplier effect on the digital development and thus influence high quality economy, which will be an indirect examination of digital development as a mechanism from a policy angle. Second, the sample size of the provincial studies was small, so future studies should be conducted using a greater amount of municipal level data to draw general conclusions from. Moreover, it can also serve as an empirical reference for countries whose economies are transitioning from rapid growth.

## Supporting information

S1 File Dataset. (XLSX)

## References

[1] Chau H, Bana SH, Bouvier B, Frank MR. Connecting Higher Education to Workplace Activities and Earnings. PLOS ONE. 2023;18(3):e0282323. doi: 10.1371/journal.pone.0282323 36920887 PMC10016720

[2] Van Hiel A, Van Assche J, De Cremer D, Onraet E, Bostyn D, Haesevoets T, et al. Can Education Change the World? Education Amplifies Differences in Liberalization Values and Innovation Between Developed and Developing Countries. PLOS ONE. 2018;13(6):e0199560. doi: 10.1371/journal.pone.0199560 29928058 PMC6013109

[3] Qin Y, Wang XK. The Expansion of Higher Education and the Economic Growth in China: an Empirical Analysis Based on the Provincial Panel Data. Journal of Macro-quality Research. 2017;5(03):49–61.

[4] Schultz TW. Investment in Human Capital. The American Economic Review. 1961;51:1–17.

[5] Nica E, Popescu GH. The Economics of Higher Education in the United States. Knowledge Horizons—Economics. 2014;6(1):87–90.

[6] Glaeser EL, Scheinkman J, Shleifer A. Economic Growth in A Cross-section of Cities. Journal of Monetary Economics. 1995;36(1):117–143.

[7] Papageorgiou C. Distinguishing Between the Effects of Primary and Post‐primary Education on Economic Growth. Review of Development Economics. 2003;7(4).

[8] He HQ. High Quality Development of Economy Driven by Science and Technology Finance: Realistic Predicament and Path Choice. Social Sciences in Guangxi. 2018(12):90–95.

[9] Herbertsson TT. Accounting for Human Capital Externalities with An Application to the Nordic countries. European Economic Review. 2003;47(3):553–567.

[10] Harrod RF, Denison EF. Why Growth Rates Differ: Postwar Experience in Nine Western Countries. Economica. 1969;36(143):323.

[11] Agasisti T, Bertoletti A. Higher Education and Economic Growth: A Longitudinal Study of European regions 2000–2017. Socio-Economic Planning Sciences. 2022;81.

[12] Oancea B, Pospisil R, Dragoescu RM. Higher Education and Economic Growth: A Comparison Between the Czech Republic and Romania. Prague Economic Papers. 2017;26(4):467–486.

[13] Zhu TT, Peng HR, Zhang YJ. The Influence of Higher Education Development on Economic Growth: Evidence from Central China. Higher Education Policy 2018;31(2):139–157.

[14] Bhorat H, Cassim A, Tseng D. Higher Education, Employment and Economic Growth: Exploring the Iinteractions. Development Southern Africa. 2016;33(3):312–327.

[15] Dominguez JFC. Higher Education, Regional Growth and Cohesion: Insights from the Spanish Case. Regional Studies. 2021;55(8):1403–1416.

[16] Horii R, Kitagawa A, Futagami K. Availability of Higher Education and Long-Term Economic Growth. Japanese Economic Review. 2008;59(2):156–177.

[17] Bouchakour R, Saad M, Guermat C. Higher education teaching and training system and economic performance: an empirical investigation. Journal of Education and Work. 2019;32(5):500–517.

[18] Allais S. Towards measuring the economic value of higher education: lessons from South Africa. Comparative Education. 2017;53(1):147–163.

[19] Lucas RE. On the Mechanics of Economic Development. Journal of Monetary Economics. 1988;22(1):3–42.

[20] Zhou GY, Luo SM. Higher Education Input, Technological Innovation, and Economic Growth in China. SUSTAINABILITY. 2018;10(8).

[21] Romer PM. Human capital and growth: Theory and Evidence. Carnegie-Rochester Conference Series on Public Policy. 1990;32(1):251–286.

[22] Qi D, Ali A, Li T, Chen YC, Tan JC. An Empirical Analysis of The Iimpact of Higher Education on Economic Growth: The Case of China. Frontiers in Psychology. 2022;13. 10.3389/fpsyg.2022.959026 PMC9435527 36059744

[23] Akpan AI. The Impact of Urbanization and Institutions of Higher Education on Houston Texas’ Third Ward Community. Journal of Applied Sciences and Environmental Management. 2006;10(2):29–36.

[24] Du MY, Ren SY. Does the digital economy promote industrial green transformation? Evidence from spatial Durbin model. Journal of Information Economics. 2023;1:1–17.

[25] Zhanbayev R, Bu WC. How does digital finance affect industrial transformation? Journal of Information Economics. 2023;1:18–30.

[26] Mao J, Liu JM, Liu ZY. Tax Effect of Digital Economy Development in China: The Policy Effect and Transmission Mechanism. Journal of Information Economics. 2023;1:47–58.

[27] Zhao TT, Jiao FY, Wang ZW. Digital economy, entrepreneurial activity, and common prosperity: Evidence from China. Journal of Information Economics. 2023;1:59–71.

[28] Yuan XL, Li CJ, Li ZP. Present Situation, Perplexity and Prospect of High-Quality Development of Chinese Economy. Journal of Xi’an Jiaotong University(Social Sciences). 2019;39(06):30–38 https://kns.cnki.net/kcms/detail/61.1329.C.20191008.20191718.20191006.html.

[29] Zhao JB, Shi D, Deng Z. A Framework of China’s High-quality Economic Development. Research on Economics and Management. 2019;40(11):15–31 https://kns.cnki.net/kcms/detail/11.1384.F.20191104.20190845.20191002.html.

[30] He DM, Liu P. Population Aging, Manufacturing Transformation and Upgrade, and High-quality Economic Development: Based on Mediating Effect Model. Research on Economics and Management. 2020;41(01):3–20 https://kns.cnki.net/kcms/detail/11.1384.F.20200102.20201626.20200001.html.

[31] Ma XW, Xu JW. Impact of Environmental Regulation on High-Quality Economic Development. FRONTIERS IN ENVIRONMENTAL SCIENCE. 2022;10.

[32] Zhou B, Zeng XY, Jiang L, Xue B. High-quality Economic Growth under the Influence of Technological Innovation Preference in China: A Numerical Simulation from the Government Financial Perspective. Structural Change and Economic Dynamics. 2020;54:163–172.

[33] Prasetyo PE, Kistanti NR. Human Capital, Institutional Economics and Entrepreneurship as a Driver For Quality & Sustainable Economic Growth. Entrepreneurship and Sustainability Issues. 2020;7(4):2575–2589.

[34] Abanyam NL, David F, Ibrahim YD. The Role of Higher Education in Sustainable Economic Development in Nigeria: A Functionalist Theretical Perspective Analysis Sapientia Global Journal of Arts, Humanities and Development Studies. 2020;3(2):276–284.

[35] Min WF. Research on the Mechanism of How Education Promotes Economic Growth. Peking University Education Review. 2017;15(03):123–136+190–191

[36] Bygstad B, Øvrelid E, Ludvigsen S, Dæhlen M. From Dual Digitalization to Digital Learning Space: Exploring the Digital Transformation of Higher Education. Computers & Education. 2022;182:104463.

[37] Telukdarie A, Munsamy M, Ieee. Digitization of Higher Education Institutions. 2019 IEEE International Conference on Industrial Engineering and Engineering Management (IEEM)2019. p. 716–721.

[38] Hofman A, Aravena C, Aliaga V. Information and Communication Technologies and Their Impact in the Economic Growth of Latin America, 1990–2013. Telecommunications Policy. 2016;40(5):485–501.

[39] Brodny J, Tutak M. Analyzing the Level of Digitalization Among the Enterprises of the European Union Member States and Their Impact on Economic Growth. Journal of Open Innovation: Technology, Market, and Complexity. 2022;8(2):70.

[40] Wu QJ, Chen X, Wang F, Yang WG. Whether AI will Bring Mass Unemployment? Based on Calculation of AI Technology, Economic Benefits, and Employment of E-commerce Platforms. Shandong Social Sciences. 2019(03):73–80.

[41] Li HG. Digital New Infrastructure, Spatial Spillover and High-quality Economic Development. Inquiry into Economic Issues. 2022(06):28–39

[42] Liu Y, Yang Y, Li H, Zhong K. Digital Economy Development, Industrial Structure Upgrading and Green Total Factor Productivity: Empirical Evidence from China’s Cities. International Journal of Environmental Research and Public Health. 2022;19(4):2414. doi: 10.3390/ijerph19042414 35206606 PMC8872123

[43] Ren BP, Song XC. The Judgment and Realization Path of High-Quality Development of China’s Economy under the Guidance of New Development Concept. Economic Review Journal. 2020(06):45–54+42.

[44] Ma D, Zhu Q. Innovation in Emerging Economies: Research on the Digital Economy Driving High-quality Green Development. Journal of Business Research. 2022;145:801–813.

[45] Facchini F, Seghezza E. Public Spending Structure, Minimal State and Economic Growth in France (1870–2010). Economic Modelling. 2018;72:151–164.

[46] Castro C, Lopes C. Digital Government and Sustainable Development. Journal of the Knowledge Economy. 2022;13(2):880–903. 10.1007/s13132-021-00749-2 PMC7896552 40477435

[47] Pugh R, Soetanto D, Hamilton E, Gibbons A, Ronan N, Jack S. Nuancing the Roles of Entrepreneurial Universities in Regional Economic Development. Studies in Higher Education. 2022;47(5):964–972.

[48] Wang X, Liu J. China’s Higher Education Expansion and the Task of Economic Revitalization. Higher Education. 2011;62(2):213–229.

[49] Hu SP. China’s Higher Education over the Past 70 Years: Scale, Quality, Innovation and Prospect. Fudan Education Forum. 2019;17(05):5–8+20.

[50] Ergen H, Cakioglu V. The Expansion of Higher Education in Turkey (1980–2016): Some Economic Implications. YUKSEKOGRETIM DERGISI. 2018;8(2):207–233.

[51] Abbas A, Hosseini S, Nunez JLM, Sastre-Merino S. Emerging Technologies in Education for Innovative Pedagogies and Competency Development. Australasian Journal of Educational Technology. 2021;37(5):1–5.

[52] Zhavoronok A, Kholiavko N, Dubyna M, Djakona A, Lavrov R. The Higher Education Adaptability to the Digital Economy. Bulletin the National Academy of Sciences of the Republic of Kazakhstan. 2020;4(36):294–306.

[53] Kholiavko N, Popelo O, Melnychenko A, Derhaliuk M, Grynevych L. The Role of Higher Education in the Digital Economy Development. REVISTA TEMPOS E ESPACOS EDUCACAO. 2022;15(34):1–14.

[54] Sun JH, Wei L. Can the Development of Higher Education be Transformed into the Growth Point of Regional Economy? Jiangsu Higher Education. 2022;No.261(11):10–18.

[55] Wang HJ, Lan ZM. College Resources Allocation, Space Spillover and Urban Innovation. Inquiry into Economic Issues. 2022;No.476(03):44–55.

[56] Nie J, Xin SB. Analysis of Quality Differentiation Measurement in Higher Education in China and Its Effect on Regional Economic Growth. China Soft Science. 2018(11):58–65.

[57] Martín-Núñez JL, Ar AY, Fernández RP, Abbas A, Radovanović D. Does Intrinsic Motivation Mediate Perceived Artificial Iintelligence (AI) Learning and Computational Thinking of Students During the COVID-19 Pandemic? Computers and Education: Artificial Intelligence. 2023;4:100128.

